# Incidence of Osteoradionecrosis and Dental Implant Survival in Patients With Irradiated Head and Neck Malignancies: A Systematic Review and Meta-Analysis

**DOI:** 10.7759/cureus.116387

**Published:** 2026-09-16

**Authors:** Nirmalya Chatterjee, Amrita Ghosh, Arka Swarnakar, Snigdho Das

**Affiliations:** 1 Department of Prosthodontics, Burdwan Dental College and Hospital, Burdwan, IND; 2 Department of Conservative Dentistry and Endodontics, Dr. R. Ahmed Dental College and Hospital, Kolkata, IND; 3 Department of Prosthodontics, Kothiwal Dental College and Research Centre, Moradabad, IND; 4 Department of Conservative Dentistry, Ramakrishna Sarada Mission Matri Bhavan, Kolkata, IND

**Keywords:** dental implants, head and neck neoplasms, osteoradionecrosis, radiotherapy, survival rate, treatment outcome

## Abstract

This systematic review and meta-analysis assessed dental implant survival and osteoradionecrosis (ORN) after implant placement in irradiated patients with head and neck malignancies. Fourteen observational studies fulfilled the eligibility criteria, and 11 were suitable for a quantitative assessment of implant survival. Overall, dental implants showed good survival in irradiated tissues, but there was a wide variation among studies. Survival tended to be higher in the mandible than in the maxilla, but the difference between anatomical sites was not statistically significant. ORN following implant placement was uncommon at both the patient and implant levels. These findings suggest that implant-supported rehabilitation can be a viable option for appropriately selected irradiated patients; however, the predominance of retrospective evidence and substantial between-study heterogeneity limit the certainty of the findings. Well-designed prospective studies with standardized reporting are needed to better define factors influencing implant survival and ORN risk.

## Introduction and background

Head and neck malignancies form a major health burden in India, with lip and oral cavity cancer contributing to a significant proportion of newly diagnosed cancers, especially among males [1]. The treatment of these malignancies often entails surgery, radiotherapy (RT), chemotherapy, or combinations of these modalities, which may have major functional and anatomical repercussions influencing subsequent oral rehabilitation [1].

One of the most severe consequences after irradiation is osteoradionecrosis (ORN) of the jaws, which is defined as devitalized irradiated bone with poor healing in the absence of chronic or recurring malignancy [2]. The pathogenesis of ORN has evolved from the classic paradigm of radiation-induced hypoxia, hypocellularity, and hypovascularity to contemporary theories of radiation-induced fibroatrophic changes, endothelial injury, impaired cellular turnover, and impaired tissue remodeling [2]. These changes affect the capacity of regeneration and healing of irradiated tissues and contribute to the formation and persistence of ORN [2]. ORN development is complex, with documented risk factors including radiation dosage, smoking, poor oral hygiene, periodontal disease, systemic comorbidities, and invasive dentoalveolar operations [2].

Dental implants are an essential alternative in prosthetic rehabilitation after treatment of head and neck malignancies; however, the insertion of implants in irradiated bone presents distinct biological and clinical problems [3]. Radiation-induced changes can have a negative impact on osseointegration and implant longevity, and the effects can differ depending on the radiation dose and anatomical area [3]. Another key aspect is the timing of implant placement following RT, with prior research indicating that the gap between RT and implant placement may affect implant failure [4]. However, systematic evidence suggests that acceptable survival can be achieved with implant rehabilitation in irradiated patients, but ORN is an important adverse consequence [5].

Despite the existing evidence, there is still ambiguity regarding implant survival and ORN after implant placement in irradiated patients. The variability documented in the literature is large [3-5]. Thus, this systematic review and meta-analysis were designed to evaluate (1) the incidence of ORN after dental implant placement and (2) the survival rate of dental implants placed in patients with head and neck malignancies after radiation.

## Review

Methods

Preferred Reporting Items for Systematic Reviews and Meta-Analyses (PRISMA) guidelines guided the conduct of this review, which was registered accordingly (registration number: CRD420251103534) (Figure 1) [6].

**Figure 1:**
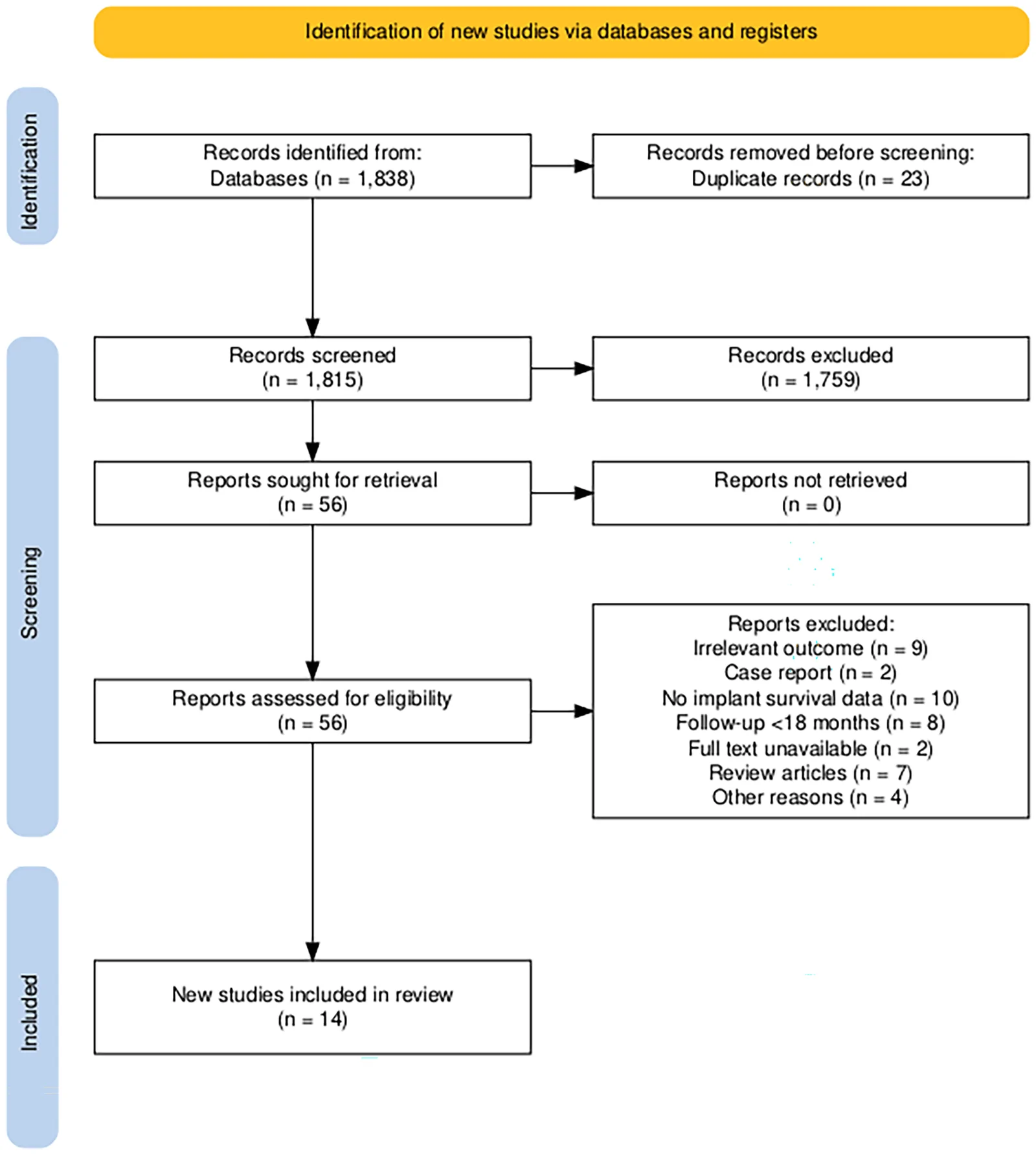
Flow diagram of study selection as per PRISMA guidelines.

Searches were conducted in the following electronic databases: PubMed/MEDLINE, Web of Science, and ScienceDirect. A manual search was also conducted by screening the reference lists of included studies and relevant review studies to identify additional eligible studies. The search was conducted to address the question: What is the survival rate of dental implants placed in patients with head and neck malignancy undergoing RT before or after implant placement? Additionally, the rate of ORN after implant placement in irradiated jaw was also assessed. Medical Subject Headings (MeSH) descriptors together with free‑text terms were employed in the search strategy, including “Dental Implants”, “Radiotherapy”, “Head and Neck Neoplasms”, and “ORN” combined using Boolean operators (AND/OR) (see Appendices). The review question was structured using a Population-Exposure-Outcome (PEO) framework (Table 1).

**Table 1:** PEO framework of the systematic review.

PEO Component	Description
Population (P)	Patients with head and neck malignancies who received radiotherapy and underwent dental implant placement
Exposure (E)	Dental implant placement in irradiated maxillary and/or mandibular bone
Outcome (O)	Dental implant survival and incidence of osteoradionecrosis (ORN)

Eligibility Criteria

The review encompassed English publications dated January 1, 2000, through July 30, 2025. Clinical data (defined as patient- or implant-level outcomes following dental implant implantation, including implant survival, failure, or ORN) from observational and interventional studies were included. Reviews, in vitro and animal research, and studies with no relevant outcome data were removed. We included studies of patients with head and neck cancers who received RT and underwent dental implant placement. There were no restrictions on the kind of RT (including conventional RT, cobalt treatment, or intensity-modulated RT (IMRT)). Both initial (at the time of tumor removal) and secondary (after completion of oncologic treatment) implant placements were included. Investigations with less than 18 months of follow‑up were omitted to allow adequate evaluation of implant survival and ORN. Implant placement in grafted bone was not analyzed separately, though studies comparing grafted and native bone were included when relevant. Two reviewers (CN, GA) independently screened studies; conflicts were adjudicated by a third (DS).

Data Extraction

Initially, Microsoft Excel (Microsoft Corp., Redmond, WA, USA) was used to curate and arrange all the data (full text and included studies). Data extracted included author details, study design, sample size, radiation dose, implant timing (primary/secondary), and the reported interval between completion of RT and implant placement, where available, along with the anatomical location (maxilla/mandible), ORN definition, follow-up duration, implant survival, and ORN incidence (Table 2). The ORN incidence was analyzed at the patient level and the implant level individually. The patient-level ORN incidence was defined as the percentage of patients that acquired ORN after implant placement over the total number of patients assessed. The implant-level ORN incidence was defined as the percentage of implants associated with ORN over the total number of implants assessed. Separate analyzes were performed for the results, as each patient could have more than one implant.

**Table 2:** Comprehensive synthesis table: dental implant survival in irradiated head and neck cancer patients. * Study-reported Kaplan-Meier/cumulative survival estimate. Crude irradiated-subgroup data were used for meta-analysis where available; studies without extractable crude data were retained only in the qualitative synthesis. RT: radiotherapy; Gy: Gray; ORN: osteoradionecrosis; Dmean: mean radiation dose; QoL: quality of life; NR: not reported/quantifiable from the available study data

Sl. No.	Author (Year)	Country	Study Design	Sample (Implants/Patients)	Follow-Up Duration	RT Dose (Gy)	Implant Timing	RT-to-Implant Interval	Jaw Site Distribution	Implant Survival (%)	ORN Incidence/Notes	Key Findings
1	Visch et al., 2002 [7]	The Netherlands	Prospective longitudinal cohort study	446/130	Up to 14 yrs	40-70	Post-RT (secondary)	NR	Maxilla: 108; Mandible: 338	382/446 (85.7%) crude survival	Not reported	Survival lower when RT >50 Gy or with bone resection; mandible > maxilla.
2	Buddula et al., 2011 [8]	United States	Retrospective cohort study	271/48	3 yrs (median)	≥50	Post-RT	NR	Maxilla: 62; Mandible: 209	238/271 (87.8%) crude survival	Not reported	No significant difference between native vs grafted bone; failures more in maxilla/posterior.
3	Mancha de la Plata et al., 2012 [9]	Spain	Retrospective cohort	225/30 (irradiated)	Mean: 45 months (6-96)	50-70	Post-RT (secondary)	Mean: 33.4 ± 18.1 months (range 12-96 months)	Maxilla: 94; Mandible: 131	92.6% at 5 yrs*	ORN occurred before subsequent implant placement in affected patients; therefore not included as incident post-implant ORN	Higher implant loss in patients with a history of ORN; 5-yr survival ≈48% in ORN subgroup.
4	Korfage et al., 2014 [10]	Netherlands	Prospective cohort	524/164 (318 irradiated/206 non-irradiated)	Up to 14 years	Not specified	Primary (before postoperative RT)	Not applicable	Mandibular interforaminal overdentures predominant	291/318 (91.5%) crude survival in irradiated implants	5/100 irradiated patients; 10/318 irradiated implants	27/318 losses in irradiated vs 1/206 non-irradiated (excluding tumor resections).
5	Pompa et al., 2015 [11]	Italy	Retrospective observational cohort study	168/34	5 yrs	<50 (12 irradiated patients)	Post-RT	12-89 months; minimum interval 12 months	Maxilla: 72; Mandible: 96	39/51 (76.5%) crude survival in irradiated subgroup	Not reported	RT and early loading (<6 mo) → lower survival; delayed loading favorable.
6	Rana et al., 2016 [12]	India	Retrospective clinical cohort study	162/46	6 yrs	Stratified as <50 vs >50	Post-RT	Mean 15 months (range 6-24 months)	Maxilla: 70; Mandible: 92	110/162 (67.9%) crude survival	Not reported	Failures increased with higher dose; no significant difference between jaws; survival multifactorial.
7	Flores-Ruiz et al., 2018 [13]	Spain	Observational retrospective cohort study	106/17 overall; 78 RT implants	5 yrs	Not specified	Post-RT	NR	RT implants: Maxilla 29; Mandible 49	85.9% in RT-treated group*	Not reported	Lower survival was associated with treatment/site-related factors; exact RT-specific crude survival numerator was not available.
8	Desoutter et al., 2018 [14]	France	Monocentric retrospective cohort study	40/18	10 yrs	50-66	Post-RT	Mean 44.6 months (range 6-183 months)	Mandible only	32/40 (80.0%) crude survival	1/18 patients; 1/40 implants	Failures clustered when dose >55 Gy; ORN risk increased with soft-tissue trauma.
9	Papi et al., 2019 [15]	Italy	Retrospective cohort	113/32	Mean±SD (range): 25.5±3.4 (25-30) months	60-70	Post-RT	NR	Maxilla: 35; Mandible: 78	107/113 (94.7%) crude survival	1/32 patients; implant-level count not established	No difference in survival.
10	Laverty et al., 2019 [16]	United Kingdom	Retrospective service-evaluation cohort study	779/167 overall; 525 RT implants in 105 patients	3-5 yrs (median 38 months)	Not specified	Mixed (primary + secondary)	NR; exact RT-to-implant interval not reported	Not separated	499/525 (95.0%) crude survival in RT subgroup; 95.5% at 5 yrs overall*	1/105 RT patients; 1/525 RT implants	RT did not significantly affect survival; failures higher in reconstructed bone.
11	Schiegnitz et al., 2021 [17]	Germany	Retrospective long-term cohort study	711/164 overall; 291 irradiated secondary-placement implants used in meta-analysis	Up to 10 yrs	50-70	Primary + Secondary	Mean 43.6 months for secondary implant placement	Maxilla: 234; Mandible: 477	260/291 (89.3%) crude survival in irradiated secondary-placement subgroup; 87.3% at 5 yrs and 80.0% at 10 yrs overall*	Not reported	Augmented + irradiated bone → lowest survival (64%); QoL improved.
12	Lee et al., 2022 [18]	Republic of Korea	Retrospective dosimetric cohort study	90/27	3 yrs	Dmean <38 vs ≥38	Post-RT	Median 28.2 months (range 1.7-121.5 months)	Maxilla + Mandible	74/90 (82.2%) crude survival; 79.6% 3-yr cumulative survival*	Not included in ORN meta-analysis because an incident RT-specific ORN event count was not established	Dmean = independent prognostic factor; no failures if Dmean <38 Gy; grafted bone ↑ risk (OR = 32.9).
13	Schweyen et al., 2022 [19]	Germany	Retrospective cohort	328 implants/71 implant patients overall; 65 implants in non-RT patients	Mean 22.9 months	Mean 64.6	Post-RT	NR	Maxilla + Mandible	89.9% at 5 yrs overall*	Two late ORN-associated failures reported; RT-specific incidence could not be established	Mixed RT/non-RT cohort; exact irradiated-specific crude survival numerator was unavailable.
14	Lee et al., 2023 [20]	Republic of Korea	Retrospective analytical cohort study	83/23	10 yrs (2010-2020)	Average 56.97	Post-RT	Mean 6.46 years (~77.5 months)	Maxilla: 43; Mandible: 40	76/83 (91.6%) crude survival	Not reported	Survival lower in irradiated maxilla; cancer type and site significant; dose not.

The interval between the end of radiation and the implantation of the implant was reported inconsistently in the included trials. Where data were available, the interval between completion of radiotherapy and implant placement varied from around six months to several years, with the mean or median interval differing greatly between studies. In some studies, the main implant was placed before postoperative RT while in others, it was placed after RT. We did not deem a pooled statistical evaluation of the influence of implant-placement time appropriate due to the use of varied measures for reporting timing and the lack of stratified implant survival or failure outcomes according to comparable RT-to-implant intervals in most studies.

Statistical Analysis

The collected data were tabulated using Microsoft Excel. Meta-analysis was performed using RStudio version 2025.09.0 (Build 387; Posit Software, PBC, Boston, Massachusetts, USA).

Risk of bias was assessed using the “Quality in Prognosis Studies” (QUIPS) tool. The robvis application [21] was used to create traffic-light and summary plots for visualization. The strength of evidence was assessed using the Grading of Recommendations Assessment, Development and Evaluation (GRADE) framework, modified for prognostic contexts.

Pooled implant survival and ORN outcomes were estimated using single-arm meta-analysis of proportions. Proportions were logit-transformed (PLOGIT), and inverse-variance random-effects models with restricted maximum likelihood (REML) estimation were used to account for within- and between-study variation. Subgroup analysis was performed according to jaw location (maxilla versus mandible). Statistical heterogeneity was assessed using the I^2^ statistic and τ^2^, with I^2^ >50% indicating substantial heterogeneity. Potential sources of heterogeneity, including implant timing, radiation dose, jaw involvement, and grafting, were examined qualitatively. Funnel plot assessment and Egger’s regression test were performed for the overall implant survival analysis, which included at least 10 studies. Statistical significance was set at P<0.05.

Results

Study Characteristics

Fourteen studies published between 2002 and 2023 were included in the systematic review. Eleven studies provided extractable crude survival data from irradiated implant groups and were included in the overall implant survival meta-analysis, comprising 2,390 implants. Jaw-specific data suitable for subgroup meta-analysis were available from five study estimates for the maxilla and seven for the mandible, comprising 318 and 1,115 implants, respectively. Four studies contributed to the patient-level ORN analysis, whereas three studies contributed to the implant-level ORN analysis. Most included studies were retrospective, and radiation doses generally ranged from approximately 50 to 70 Gy. Both maxillary and mandibular implants were evaluated, with a greater proportion of implants placed in the mandible.

Risk of Bias Evaluation

The methodology quality was deemed acceptable, with most studies showing low risk of bias in participation and outcome measurement. Moderate bias risk was noted in confounding and prognostic factor measurement due to inconsistent reporting of radiation doses and implant timing. Study attrition reporting varied. Ultimately, four studies had a low risk of bias, while the others were classified as moderate, with no studies rated as high risk (Figures 2a-2b).

**Figure 2:**
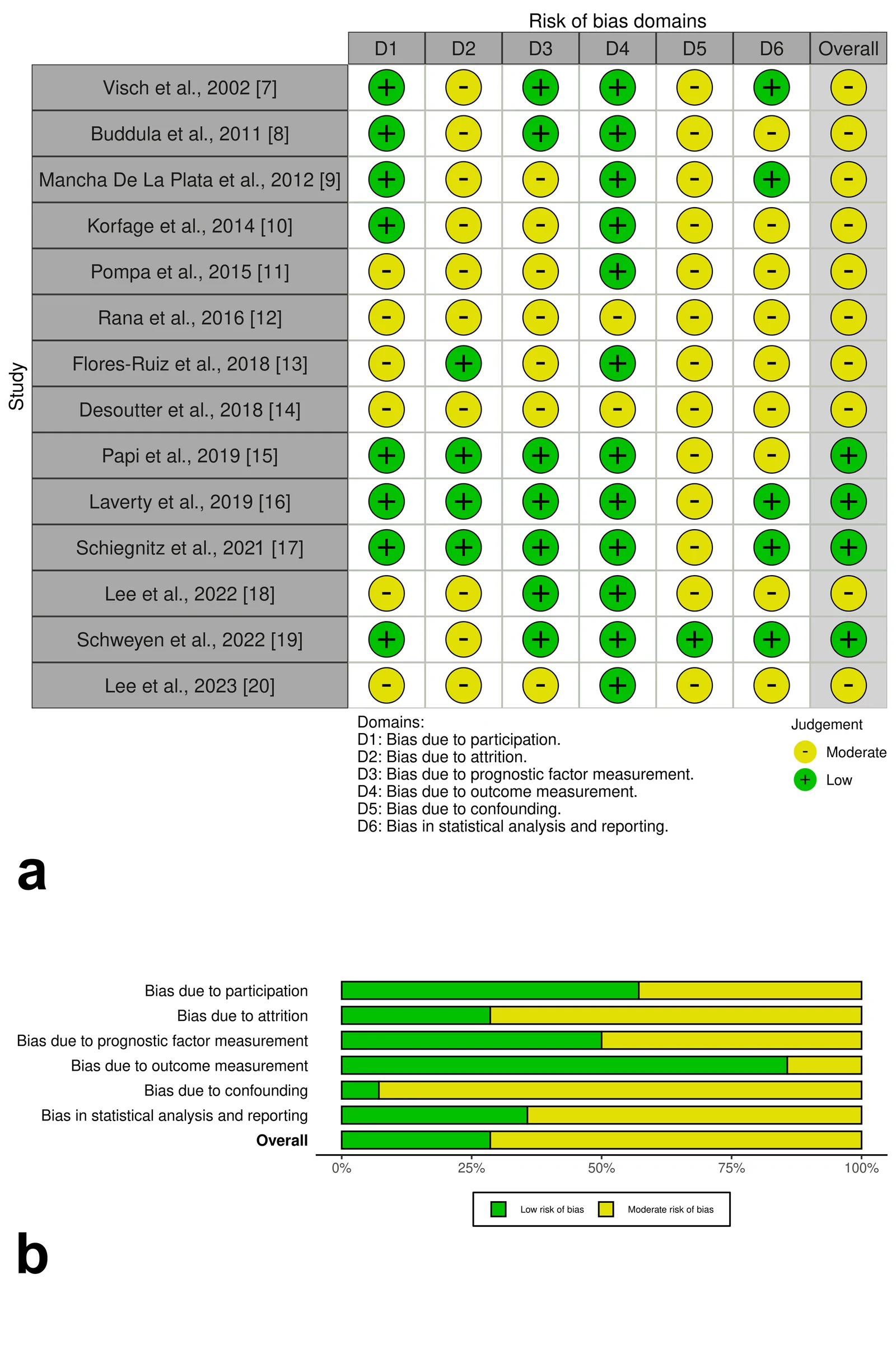
Risk of bias assessment using the QUIPS tool: (a) traffic-light plot showing domain-wise risk of bias; and (b) summary plot showing overall risk of bias across studies. Studies included [7-20] QUIPS: Quality in Prognosis Studies

GRADE Assessment

The strength of evidence for implant survival was graded as moderate, considering the observational nature of included studies and variability across studies. The certainty of evidence for ORN incidence was graded as low because of limited event reporting and imprecision.

Quantitative Synthesis: Overall Implant Survival

The pooled survival proportion of dental implants in irradiated patients was 0.87 (95% CI: 0.82-0.91) using a random-effects model. Significant heterogeneity was observed across studies (I^2^=89.7%; τ^2^=0.4106; P<0.0001), indicating considerable variability among the included studies (Figure 3).

**Figure 3:**
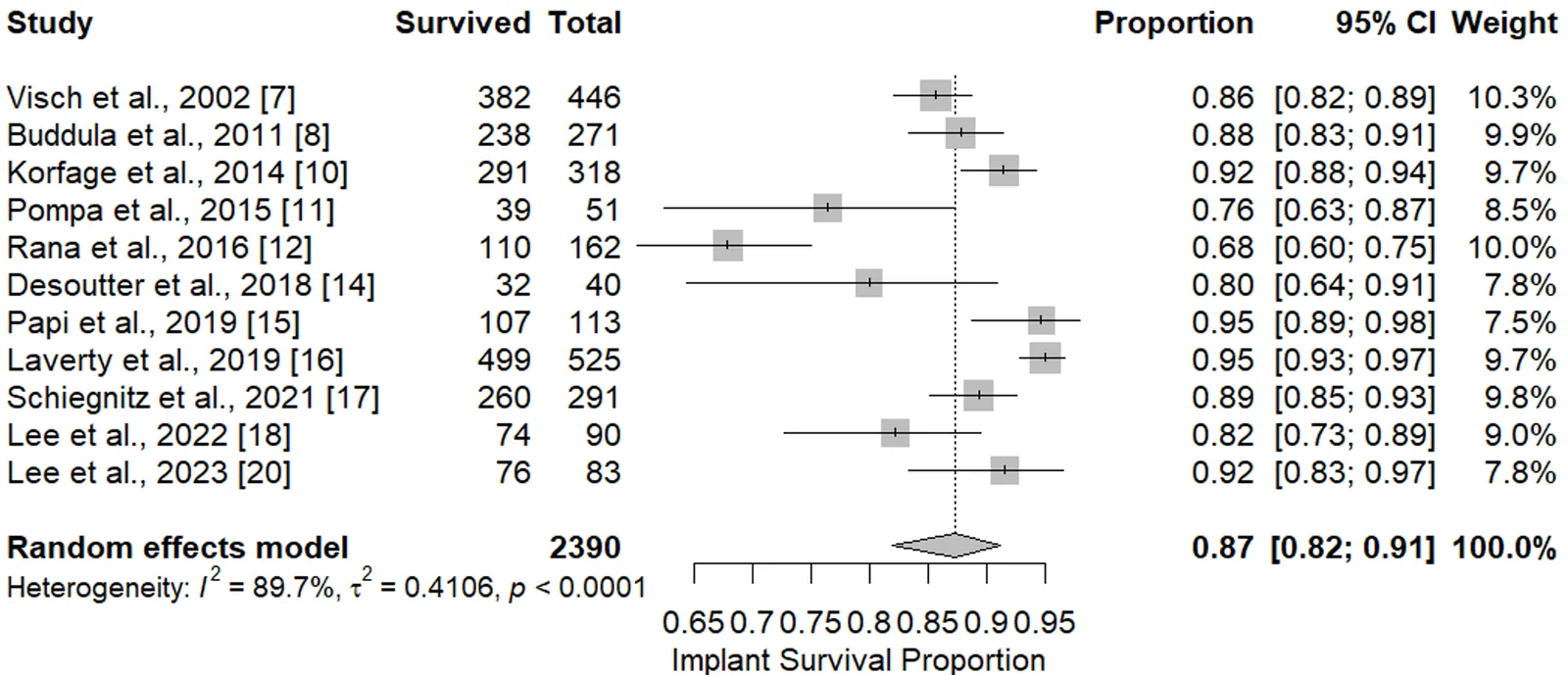
Forest plot showing pooled implant survival proportion using a random-effects REML model. Studies included [7,8,10-12,14-18,20] REML: restricted maximum likelihood

Publication bias was assessed using funnel plot inspection and Egger’s regression test. The funnel plot did not demonstrate marked asymmetry, and Egger’s regression test was non-significant (t=0.44, df=9, P=0.6685), providing no statistical evidence of funnel plot asymmetry. Nevertheless, this finding should be interpreted cautiously given the limited number of studies (Figure 4).

**Figure 4:**
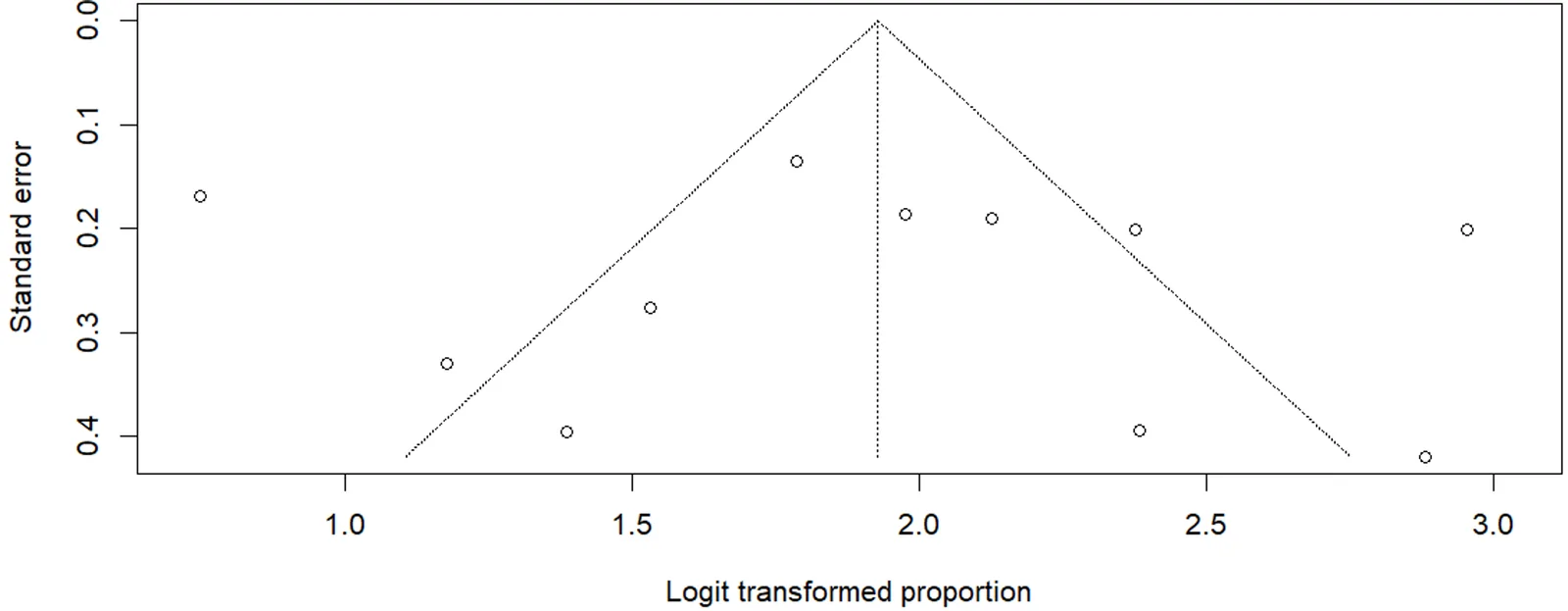
Funnel plot assessing publication bias for overall implant survival.

Subgroup Analyses by Jaw

Jaw-specific subgroup analysis included five study estimates for the maxilla and seven for the mandible. The pooled implant survival proportion was 0.80 (95% CI: 0.64-0.90; I^2^=72.6%) in the maxilla and 0.88 (95% CI: 0.80-0.94; I^2^=89.7%) in the mandible. Although the pooled estimate was numerically higher in the mandible, the difference between jaw subgroups was not statistically significant (P=0.2051) (Figure 5).

**Figure 5:**
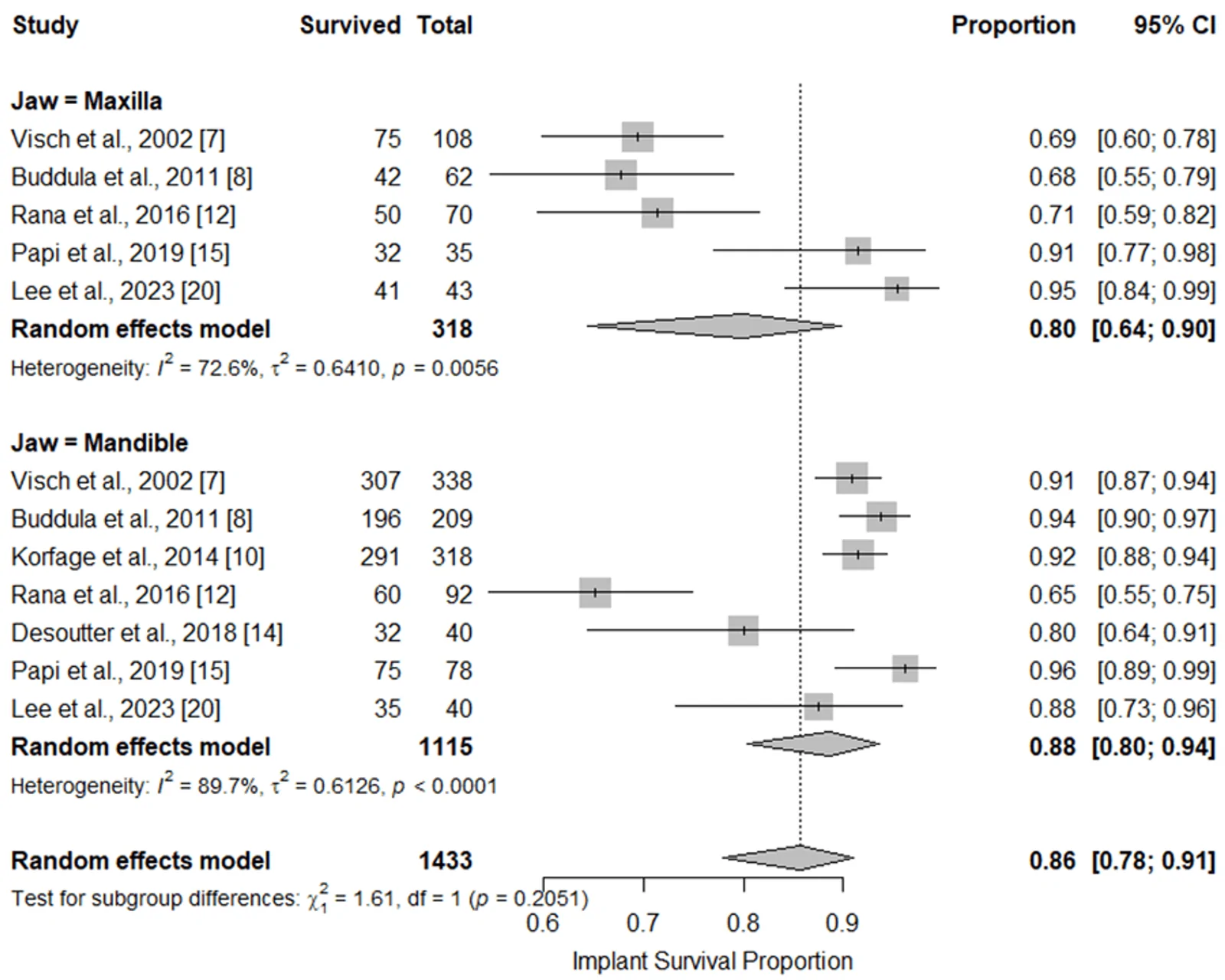
Forest plot showing implant survival in the maxilla and mandible subgroups. Studies included [7,8,10,12,14,15,20]

Incidence of ORN at Patient and Implant Levels

The pooled patient-level incidence of ORN was 0.039 (95% CI: 0.019-0.076), with no observed heterogeneity (I^2^=0.0%) (Figure 6).

**Figure 6:**
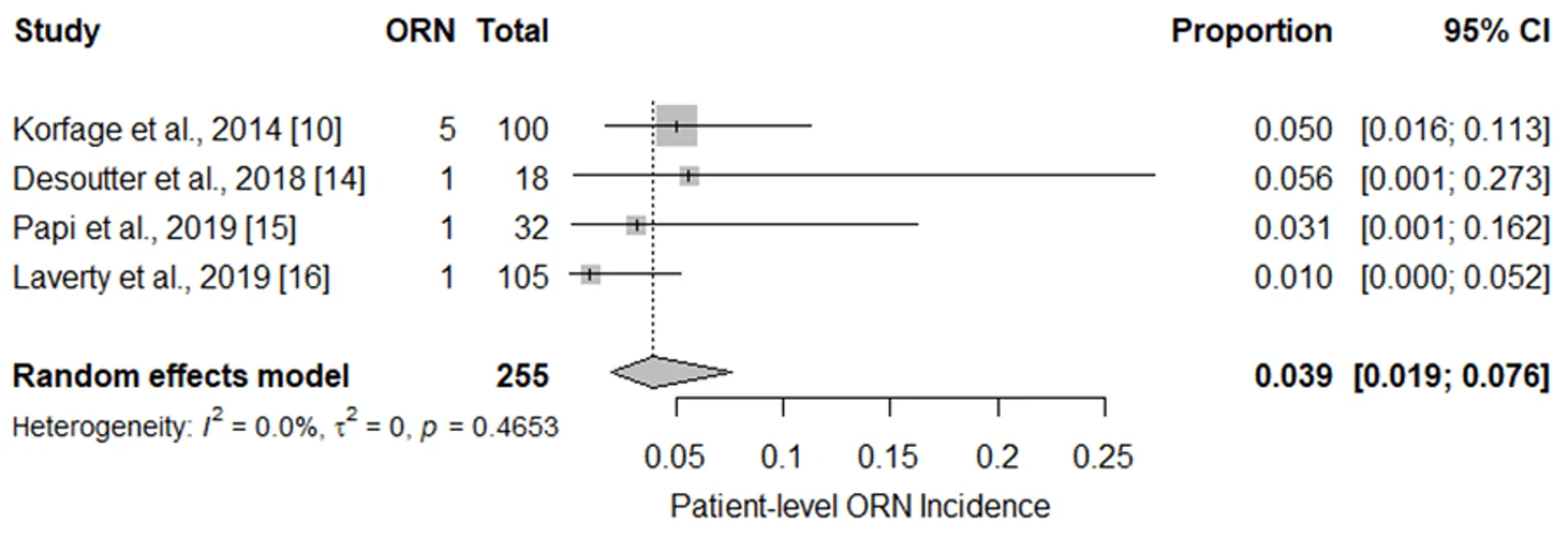
Forest plot showing pooled ORN proportion at the patient level. ORN: osteoradionecrosis Studies included [10,14-16]

The pooled implant-level incidence of ORN was 0.013 (95% CI: 0.002-0.069), with substantial heterogeneity (I^2^=72.5%; τ^2^=1.6227; P=0.0264) (Figure 7).

**Figure 7:**
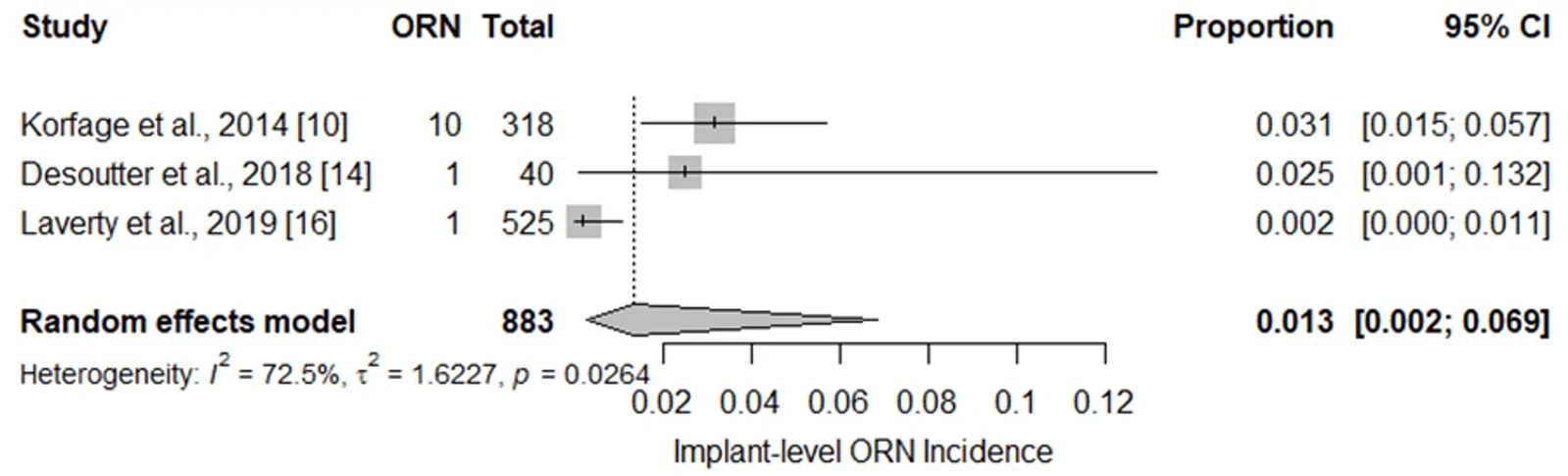
Forest plot showing pooled ORN proportion at the implant level. ORN: osteoradionecrosis Studies included [10,14,16]

Discussion

This meta‑analysis and systematic review demonstrated a relatively low incidence of ORN at both implant and patient levels, along with acceptable implant survival rates in irradiated patients. These findings suggest that, with appropriate case selection and treatment planning, implant rehabilitation in irradiated bone is feasible. However, the observed heterogeneity across studies highlights the influence of multiple clinical and treatment-related variables, necessitating cautious interpretation of pooled estimates.

ORN

ORN is devitalized, irradiated bone that does not heal in three to six months in the absence of local neoplastic disease. Definitions of ORN differed between studies, for example, length of bone exposure and diagnostic criteria, which may have influenced the incidence rates. High doses of radiation (70 Gy) are likely to be responsible for early-onset ORN (within two years), but late presentation is often caused by trauma or delayed wound healing in compromised tissue [22].

The low incidence observed in the present meta-analysis is consistent with Gorjizad et al., who reported an ORN incidence of 1.81% [5]. In the present study, ORN was uncommon at both the implant and patient levels, with pooled incidences of approximately 1.3% (95% CI: 0.2%-6.9%) and 3.9% (95% CI: 1.9%-7.6%), respectively. No heterogeneity was observed in the patient-level analysis, whereas substantial heterogeneity was present in the implant-level analysis, potentially reflecting differences in radiation exposure, implant-related factors, and follow-up characteristics.

Irradiated patients also have implant failure associated with pathological bone fractures, high occlusal loading, soft tissue problems, radiation-induced tissue damage, and bone necrosis. The pathophysiologic basis of ORN has classically been credited to radiation-induced endarteritis and decreased microcirculation resulting in hypoxic, hypovascular, and hypocellular bone [23]. This biological concern has been addressed in previous literature, and Epstein et al. warned against invasive procedures such as dental implant placement, as the risk of ORN may be lifelong [24]. Somay et al. also examined the association between the period after RT and subsequent implant loss [25]. These observations are especially pertinent to our present findings, as the research included in this review indicated a wide difference in the time of implant placement after radiation. ORN rates of 1.6% and 1.8% have been reported following implant placement, and advances in surgical techniques and adjunctive approaches such as bone morphogenetic protein-2 (BMP-2) and platelet-rich plasma (PRP) have been discussed in relation to management of irradiated tissues [26,27]. The low pooled incidence of ORN in the present analysis, however, should not be interpreted as absence of long-term risk, as previous literature suggests that spontaneous ORN occurs most commonly within two to three years of RT, while trauma-induced ORN may occur considerably later [23,28,29].

Implant Survival

Apart from ORN, soft tissue hyperplasia and failed osseointegration largely contribute to implant failure in irradiated patients. As stated by Toneatti et al. [30], implant survival outcomes among control patients who did not undergo RT were 97% (37.7 months follow-up), higher than irradiated patients, which was 91.9% (39.8 months average follow-up), which is similar to the finding of the current study, where the implant survival in irradiated cohorts was found to be approximately 87%. Across irradiated and control groups, failed osseointegration accounted for 42.9% of losses, while peri‑implantitis contributed 28.6%. In contrast to the present study, which demonstrated a pooled implant survival proportion of approximately 87% in irradiated patients, Gorjizad et al. reported substantially lower survival (approximately 42%) in irradiated individuals, with 58% implant failure [5]. Differences in radiation exposure, surgical technique, study populations, implant timing, follow-up duration, and inclusion criteria may explain this discrepancy. Such variability highlights the heterogeneity in existing evidence and underscores the need for cautious interpretation of pooled outcomes.

Factors Influencing Outcomes

Radiation dose: Although radiation dose has been suggested as a critical determinant of implant survival and ORN risk, a dose-response relationship could not be established in the present analysis due to heterogeneous reporting and lack of standardized dose stratification.

Jaw differences: The anatomical site also appears to influence implant outcomes in irradiated patients. Tanaka et al. [23] reported that the implant failure’s relative risk in irradiated bone was two to three times higher compared to non-irradiated bone, with comparatively higher survival observed in the mandible, consistent with the numerical trend observed in the present study (0.88 in the mandible versus 0.80 in the maxilla), although the subgroup difference was not statistically significant. This may be attributed to differences in bone density and biomechanical stability between the jaws.

In contrast, Dholam and Gurav [31] reported higher implant failure rates in the maxilla, despite its relatively lower susceptibility to ORN due to better vascularity and cancellous bone architecture. They further highlighted that radiation doses exceeding 45 Gray (Gy) were associated with increased implant failure.

These findings indicate that anatomical site may influence implant outcomes; however, the present meta-analysis did not evaluate jaw-specific ORN incidence.

Timing of Implant Placement and Treatment Factors

In the present review, the interval between completion of RT and implant placement was inconsistently reported across the included studies. Where quantifiable, the reported intervals varied considerably, while some studies involved primary implant placement before postoperative RT and others did not provide a precise interval. This variability in reporting is also reflected in recent evidence; Pacheco et al. noted that the RT-to-implant interval was specifically investigated in only one of the systematic reviews included in their umbrella review [32].

Some authors support implant placement immediately after tumour surgery, suggesting that early placement allows initial osseointegration prior to radiation exposure, thereby improving survival outcomes [33-35].

Some experts propose that the generally safe period for implant placement is 6-12 months after irradiation. However, the appropriate timing is still debatable, as a lengthy delay after RT may potentially be associated with increased vascular impairment and decreased healing capacity [25]. Ramalingam et al. found positive osseointegration results for dental implants implanted at least 1.5-3 months before radiation therapy [35].

Atanásio Pitorro et al. found improved survival (89.4-90%) for implants placed before RT compared with implants placed after RT (80-100%), suggesting the potential benefit of primary implant placement [36]. On the contrary, Claudy et al. showed that implants placed within 12 months after RT had a 34% higher probability of failure, suggesting that early post-RT insertion may not be desirable [4]. In their study, Schiegnitz et al. found a meaningful influence of RT on implant survival, while chemotherapy had no significant effect [17].

As per Prado-Pena et al., implant survival was higher in non-irradiated patients; however, no statistically meaningful difference was observed between implants placed before and after RT, further highlighting the inconsistency in existing evidence regarding optimal timing [37]. Additionally, Shugaa-Addin et al. reported conflicting findings, with several studies indicating no significant impact of RT on implant survival, while others demonstrated a negative effect, underscoring the heterogeneity of available evidence [38].

ORN has been reported to have an overall incidence of up to 15% in irradiated patients; however, with the advent of contemporary RT techniques such as IMRT, the incidence has reduced to approximately 0-6% in recent literature. Marx suggested that implant placement between 6-18 months following RT may be advantageous, as chronic vascular changes detrimental to healing may not yet be fully established, while the acute effects of radiation have subsided [39].

Anderson et al. proposed that implant placement either prior to RT (approximately 14 days before) or after a delayed interval of 6-24 months may yield favorable outcomes, particularly when radiation doses are ≤50 Gy [33]. These recommendations further support the importance of treatment timing and RT parameters in influencing both implant survival and the risk of ORN, although variability in clinical protocols across studies limits the ability to establish a universally optimal time frame.

The success and lifespan of an implant can be greatly influenced by patient characteristics such as smoking, diabetes, and dental hygiene, as well as the risk of ORN. These characteristics have been shown to compromise vascularity, delay healing, and increase susceptibility to infection (albeit reported inconsistently in the studies) and may worsen results in irradiated bone [40]. Moreover, the impact of different radiation modalities, in particular IMRT versus conventional RT, could not be examined because of inconsistent reporting and lack of subgroup data among the included studies, which is a critical limitation of the present research.

Moreover, the current study has a limited evaluation of publication bias, as funnel plot and Egger’s regression analysis were only performed for outcomes with 10 or more studies [41]. Furthermore, most studies in the available literature were of retrospective design, which by design inherently restricts the quality of evidence, and the conclusions should be interpreted cautiously given the risk of selection bias and lack of uniform techniques across research [42].

In this study, the pooled implant survival proportion in irradiated patients was 87%. Jaw-specific subgroup analysis yielded pooled survival proportions of 80% in the maxilla and 88% in the mandible, although this difference was not statistically significant. Factors like implant timing and radiation dose could potentially affect outcomes, but due to heterogeneity and inconsistent reporting, they could not be independently analyzed. Thus, no definitive conclusions are available regarding these variables. Future prospective studies with standardized protocols are necessary to develop clearer clinical guidelines. Despite these limitations, the findings support the viability of implant rehabilitation in irradiated patients.

## Conclusions

This systematic review and meta-analysis demonstrated favorable dental implant survival in irradiated patients with head and neck malignancies. Implant survival was higher in the mandible than in the maxilla, although the difference between anatomical sites was not statistically significant. ORN following implant placement was uncommon at both the patient and implant levels. Overall, the findings indicate that dental implant rehabilitation can achieve favorable outcomes in appropriately selected irradiated patients, although the substantial heterogeneity in implant-survival estimates warrants cautious interpretation.

## Appendices

**Table 3:** Search terms. Limits applied: Studies published between January 2000 and July 2025; English language.

Database	Search Strategy
PubMed/MEDLINE	#1 ("Head and Neck Neoplasms"[MeSH] OR "head and neck cancer" OR "oral cancer") #2 ("Radiotherapy"[MeSH] OR "radiation therapy" OR irradiated OR IMRT OR cobalt) #3 ("Dental Implants"[MeSH] OR "dental implant*") #4 ("Osteoradionecrosis"[MeSH] OR osteoradionecrosis OR ORN) #5 ("Survival Rate"[MeSH] OR survival OR failure OR "implant survival") #6 #1 AND #2 AND #3 AND (#4 OR #5)
Web of Science	#1 TS=("head and neck cancer*" OR "oral cancer*" OR "head and neck neoplasm*") #2 TS=("radiotherapy*" OR "radiation therapy" OR irradiated OR IMRT OR cobalt) #3 TS=("dental implant*") #4 TS=("osteoradionecrosis*" OR ORN OR survival OR failure) #5 #1 AND #2 AND #3 AND #4
ScienceDirect	("head and neck cancer" OR "oral cancer") AND ("radiotherapy" OR "irradiated") AND ("dental implant") AND ("osteoradionecrosis" OR survival OR failure)
Manual Search	Reference lists of included studies and relevant systematic reviews were screened to identify additional eligible studies.
